# Efficient Data-Driven Machine Learning Models for Cardiovascular Diseases Risk Prediction

**DOI:** 10.3390/s23031161

**Published:** 2023-01-19

**Authors:** Elias Dritsas, Maria Trigka

**Affiliations:** Department of Computer Engineering and Informatics, University of Patras, 26504 Patras, Greece

**Keywords:** healthcare, cardiovascular diseases, prediction, machine learning, data analysis

## Abstract

Cardiovascular diseases (CVDs) are now the leading cause of death, as the quality of life and human habits have changed significantly. CVDs are accompanied by various complications, including all pathological changes involving the heart and/or blood vessels. The list of pathological changes includes hypertension, coronary heart disease, heart failure, angina, myocardial infarction and stroke. Hence, prevention and early diagnosis could limit the onset or progression of the disease. Nowadays, machine learning (ML) techniques have gained a significant role in disease prediction and are an essential tool in medicine. In this study, a supervised ML-based methodology is presented through which we aim to design efficient prediction models for CVD manifestation, highlighting the SMOTE technique’s superiority. Detailed analysis and understanding of risk factors are shown to explore their importance and contribution to CVD prediction. These factors are fed as input features to a plethora of ML models, which are trained and tested to identify the most appropriate for our objective under a binary classification problem with a uniform class probability distribution. Various ML models were evaluated after the use or non-use of Synthetic Minority Oversampling Technique (SMOTE), and comparing them in terms of Accuracy, Recall, Precision and an Area Under the Curve (AUC). The experiment results showed that the Stacking ensemble model after SMOTE with 10-fold cross-validation prevailed over the other ones achieving an Accuracy of 87.8%, Recall of 88.3%, Precision of 88% and an AUC equal to 98.2%.

## 1. Introduction

Cardiovascular diseases (CVDs) have been the leading cause of death globally for the past 15 years. It is estimated that 17.9 million people died of cardiovascular diseases in 2019, accounting for 32% of all deaths worldwide. Cardiovascular disease statistics are appearing ominously around the world and it has been estimated that by 2030, casualties will exceed 20 million per year [1].

Specifically, cardiovascular disease is a set of diseases that affect the heart and blood vessels and they are mainly divided into coronary heart and arteries disease. The latter supplies blood to the brain that is responsible for strokes [2], while a peripheral arterial disease affects the arteries that supply blood to the extremities (legs, arms) [3]. Some other categories include disease of the muscles and valves of the heart followed by infection with a bacterium of the streptococcus family (rheumatic heart disease) [4], diseases that are congenital and due to dysgenesis of the structures of the heart and blood vessels of the circulatory system and, finally, diseases that are due to the formation of clots in the veins of the lower extremities (thigh, tibia, legs) [5], which can then be split, detached and transported to the heart and lungs (venous thrombosis and pulmonary embolism) [6,7].

Although cardiovascular diseases often occur suddenly with significant effects on a patient’s health, they actually have a long sub-clinical course without symptoms until they manifest clinically. This element is extremely important to prevent the occurrence of such diseases in the population [8]. Cardiovascular disease does not have a definite cause, such as infections caused by specific germs or viruses. However, there are several risk factors such as lifestyle and health profile promoting the disease manifestation. Given that people at risk for cardiovascular disease are more likely to have a heart attack, the prevention of its occurrence is mainly based on these factors’ modification in the population to reduce the likelihood of cardiovascular disease occurrence [9,10].

The most common well-documented risk factors for CVDs are age, gender, aggravated family history, lack of physical exercise (sedentary life), obesity, unhealthy diet and consumption of large amounts of salt, excessive alcohol consumption, smoking, hypertension, and high blood cholesterol levels [11,12]. Some other CVD-related factors are kidney disease that causes reduced kidney function, diabetes, and rheumatoid arthritis. Moreover, CVD may occur due to premature menopause in women [13,14,15]. In addition, people who sleep a lot or sometimes less than necessary and those who fall asleep at very different times are at greater risk for cardiovascular disease. The more irregular the duration of their sleep, the greater the risk of developing CVD due to a biological clock disorder that affects metabolism, blood pressure and heart rate [16,17].

It is important to note that people who belong to a high-risk group for cardiovascular occurrence (not necessarily diagnosed), and are thus at risk of having a heart attack or stroke, have a significantly higher risk of being seriously ill with COVID-19. In addition, these people are more likely to need hospitalization and intensive care unit (ICU) or even die from COVID-19 compared to people with low cardiovascular risk which, nowadays, constitutes a strong motivation for the underlying research [18,19].

According to previous studies, it is possible to estimate the likelihood of a person developing CVD based on several risk factors, based on which a doctor can guide patients with appropriate advice and interventions. Some preventative measures that could be taken by people at high risk for developing cardiovascular disease include: (i) medication with statins when the cholesterol level is higher than normal or to low blood pressure (in case it is high or slightly high), (ii) smoking cessation in case of smokers, (iii) healthy eating (by replacing saturated fats with unsaturated fatty acids and the intake of $\Omega_{6}$ polyunsaturated fats), (iv) maintaining weight and waist circumference under control, (v) reduction of alcohol consumption and (vi) systematic physical exercise [20,21,22].

Nowadays, ML contains a range of approaches and techniques that can be applied in a variety of ways to help the diagnostic and prognostic challenges facing the field of medicine. Moreover, ML techniques now enable medical researchers to detect significant diseases in a more sophisticated and accurate way. In this direction, ML plays an essential role in the early prediction of disease complications in diabetes (as classification [23,24] or regression task for continuous glucose prediction [25,26]), cholesterol [27], hypertension [28,29], hypercholesterolemia [30], chronic obstructive pulmonary disease (COPD) [31], COVID-19 [32], stroke [33], chronic kidney disease (CKD) [34], liver disease [35], hepatitis-C [36], lung cancer [37], sleep disorders [38], metabolic syndrome [39], etc.

In particular, the long-term risk prediction of CVDs will concern us in this research work. The contribution of this manuscript is three-fold.

- An essential step of the elaborated methodology is data preprocessing, consisting of data cleaning and class balancing. Data preprocessing is achieved with the SMOTE. In this way, the dataset’s instances are distributed in a balanced way allowing us to design efficient classification models and predict the occurrence of CVD.
- In the context of features analysis, three ranking methods, i.e., Gain Ratio, Random Forest and Information Gain were applied to measure their importance in the CVD class, and a statistical description of their prevalence is also presented.
- Experimental evaluation with several ML models after the use or not of SMOTE with 10-fold cross-validation evaluating and comparing them in terms of Accuracy, Recall, Precision and AUC in order to identify the most efficient for predicting the risk of an instance being diagnosed with CVD.

The rest of the paper is structured as follows. In Section 2, we describe the dataset we relied on and analyse the methodology we followed. In addition, in Section 3, we capture the obtained experimental results and evaluate the ML models’ performance. In addition, in Section 4, we briefly discuss works that aim to predict CVD using ML methods. Finally, conclusions and future directions are mentioned in Section 5.

## 2. Materials and Methods

In this section, we will present an analysis of the data features, which constitute important factors for measuring CVD risk. Furthermore, the main components of the adopted methodology are analysed, including data preprocessing, features ranking and prevalence, and ML model description, before the presentation of the evaluation results.

### 2.1. Dataset Description

Our experimental results were based on the dataset of research work [40]. In the given dataset, data cleaning (namely, removing records with missing or invalid values for the respective feature) [41] was performed and, finally, the number of participants we focused on was 6311. Each record in the dataset is described by a set of 10 attributes, along with their variables, which are given as input to machine learning models and one for the target class. These attributes are analyzed as follows:

- **Age** (years) [42]: It is the attribute that keeps the participant’s age. The age range is 30 to 65 years.
- **Gender** [43]: This attribute indicates the participant’s gender. The number of men is 2184 (34.6%), while the number of women is 4127 (65.4%).
- **BMI** (Kg/m^2^) [44]: This attribute illustrates the participant’s body mass index.
- **Systolic Blood Pressure (Sys BP)** (mmHg) [45]: This attribute illustrates the participant’s systolic blood pressure.
- **Diastolic Blood Pressure (Dias BP)** (mmHg) [46]: This attribute illustrates the participant’s diastolic blood pressure.
- **Glucose** [47]: This feature captures the participant’s glucose status. It has three categories (85.6% normal, 7.4% above normal and 7% well above normal).
- **Smoke** [48]: This attribute refers to whether the participant smokes or not. The percentage of participants who are smoking is 9.2%.
- **Alcohol Intake** [49]: This attribute refers to whether the participant consumes alcohol or not. Up to 5.4% of participants consume alcohol.
- **Physical Activity** [50]: This variable records whether the participant is physically active or not. The percentage of participants who have physical activity is 80.1%.
- **Total Cholesterol** [51]: This variable captures the participant’s total cholesterol status. It has three categories (78.1% normal, 12.7% above normal and 9.2% well above normal).
- **Cardiovascular Disease (CVD)**: This attribute refers to whether the participant suffers from cardiovascular disease or not. A total of 1944 (30.8%) of the participants suffer from cardiovascular disease.

All features, including the target class, are nominal, except for the age, BMI, and systolic and diastolic BP, which are numerical. In Table 1, we present the statistics of the variables that represent the risk factors for CVD in the unbalanced dataset. These variables capture the values of the related features, which are discriminated into numerical and nominal. For the description of numerical data, we recorded mean values and standard deviation, while for the nominal ones, we recorded the percentage prevalence of the respective categorical data.

### 2.2. Proposed Methodology for CVD Risk Prediction

The proposed methodology for CVD risk prediction includes several steps, such as data preprocessing, features ranking, feature prevalence in the balanced data, ML models’ configuration, training and evaluation.

#### 2.2.1. Data Preprocessing

Since the efficiency of ML models and, thus, the accurate identification of CVD and non-CVD instances may be impacted by the unbalanced distribution of the instances in the two classes, an oversampling method is applied. In particular, SMOTE (Synthetic Minority Oversampling Technique) [52] was applied, which, based on a 5-NN classifier, creates synthetic data [53] on the minority class. The instances in the CVD class are oversampled such that the subjects in the two classes are uniformly distributed. After the application of SMOTE, the dataset becomes balanced, the number of participants is 8734 and the class variable includes 4367 CVD and 4367 non-CVD instances.

#### 2.2.2. Features Ranking

In Table 2, we present the dataset features’ importance concerning the CVD class. Features ranking is made via the employment of three methods. The former exploits the Random Forest classifier to assign a score, the latter is based on the Information Gain method (InfoGain) and the third one is based on Gain Ratio.

As for the Random Forest, each tree calculates the importance of a feature according to its ability to increase the pureness of the leaves. The higher the increment in leaf purity, the higher the importance of the feature. This is applied for each tree, averaged among all the trees and normalized such that the sum of the importance scores is equal to 1 [54].

The InfoGain [55] estimates the worth of an attribute *F* by measuring the information gain with respect to the class variable *C*, according to the math equation InfoGain(C,F)=H(C)−H(C|F). The first term defines the entropy of the class variable *C* which can be determined as $H\left(C\right)=-\sum_{c\in V_{C}}p_{c}log_{2}\left(p_{c}\right)$, where $p_{c}$ is the probability $c\in V_{C}$ be equal to 0 (Non_CVD) and 1 (CVD), respectively. In a balanced dataset, $p_{0}=p_{1}=1/2$ maximises the entropy. In addition, the second term H(C|F) is the conditional entropy of the class variable *C* given an attribute *F*. The feature with the highest InfoGain is appropriate to be selected for a split. The Gain Ratio [56] evaluates the worth of an attribute *F* according to the formula $GR\left(C,F\right)=\frac{InfoGain(C,F)}{H(F)}$, where $H\left(F\right)=-\sum_{f\in V_{F}}p_{f}log_{2}\left(p_{f}\right)$ is the entropy of the attribute *F* and $p_{f}$ the probability of the value *f* of *F*.

Before the class balancing, it is observed that all methods agree on the ranking of the features (as shown in Table 2) except for the Random Forest that shows specific features (namely age, cholesterol and gender, alcohol intake) in reverse order. Moreover, the scores concerning alcohol intake and smoking are negative and very close to zero, which means that the features may not contribute to the models’ performance enhancement. Moreover, both InfoGain and GainRatio methods indicated systolic and diastolic blood pressure features as the most important and relevant risk factors for the occurrence of CVD before and after SMOTE. Focusing on the SMOTE case, we observe that each method has assigned a different ranking order. Since all features are contributing factors to CVD, they were considered for training and validation. Finally, the features’ importance will be reevaluated and the models will be retrained in case new (training) instances with the same features are available, thus all of them are kept.

#### 2.2.3. Features Prevalence in the Balanced Data

In this subsection, our aim is to make a qualitative and quantitative description of the data and explore the strength of association with the target CVD class. In the following, we selected to further analyze the balanced data as they will be considered to construct efficient models for CVD risk prediction.

In Table 3, we see the statistical characteristics of the numerical features in the balanced data. The mean age of participants is 53.36 years, while the minimum and maximum ages are 30 and 65 years. Moreover, the mean value of BMI is 27.48 which corresponds to the overweight category. Finally, the mean systolic and diastolic BP is 126.46 and 81.44, respectively, which relates to hypertension at stage I. It is important to mention that the statistics of the numerical features in the balanced data maintained the minimum and maximum values of the non-balanced one while an imperceptible difference was observed in the mean value and standard deviation.

In Table 4, we show the participants’ distribution in terms of the age group they belong to and their gender. In the marginal age groups, 30–34, 35–39 and 65–69, either none or only a small percentage of participants have been diagnosed with CVD (0.10% and 0.19%, respectively). A considerable part of the participants having a CVD is distributed in the age groups 50–54, 55–59 and 60–64 with proximal ratios of 12.06%, 13.57%, and 14.24%, respectively. As for gender, we see that in each gender status, the instances in the CVD class differ from the respective in the non-CVD class by absolute values 5.49% and 5.41%, respectively (namely, they are not distributed in a balanced way). Moreover, women that have been diagnosed with CVD are 38.33% prevalent in the dataset against the 11.67% of men.

In Table 5 is captured the percentage distribution of participants in each BMI category and the corresponding obesity subcategories, based on the rules of [25]. In the underweight class, a negligible portion of the participants is noticed, while most of the participants are shown in the healthy (i.e., the BMI values lie in the normal range), overweight and obese I classes. Moreover, it is observed that even if a participant has a normal BMI, they can still be diagnosed with CVD. In the current data, this is represented by a piece of 14.79%. At this point, we should recall from the relevant literature that obesity may be characterized by either BMI (also called overall weight-based) or waist circumference (WC), which denotes abdominal obesity. The existence of CVD instances in the healthy class of BMI categorization may relate to the aforementioned abdominal obesity which, however, in this study, cannot be identified since the WC feature is not available. The development of CVD is highly correlated with obesity risk factors, but WC availability could add critical information along with BMI for the correct classification of the CVD instances [57]. Hence, a limitation of the current data is the lack of the WC feature, which is a strong indicator of CVD occurrence.

Moreover, Table 5 shows the distribution of non-physically- and physically-active instances in the underlying classes. The portion of those who suffer from CVD in the two states of physical activity is 5.48% and 44.52%, correspondingly. From the results, it is observed that body exercise is not sufficient to avoid the manifestation of CVD. However, further details (which are not available here) on the type and duration of physical activity would help to interpret its role in CVD avoidance.

In Table 6, the total cholesterol and glucose levels are captured in terms of the class labels. A total of 37.29% of participants suffer from cardiovascular disease while having normal cholesterol levels at the same time. The coexistence of abnormal levels (both above and well above) of cholesterol and cardiovascular disease occurs in a total of 12.71% of participants. A similar trend is noticed in the distribution of the two classes’ instances concerning glucose levels. Furthermore, Table 7 shows the relationship between the two considered classes and alcohol consumption and smoking habits. A total of 45.65% of participants with CVD are not smokers while 48.08% of those with CVD also stated that they do not consume alcohol.

The proportion of hypertension categories with the CVD and non-CVD instances is presented in Table 8. Of the total participants, a small percentage of 3.53% and 1.32% has normal and elevated blood pressure, while they belong to the CVD class. In the hypertensive classes, an essential portion of 45.16% occurs.

**Figure 1:**
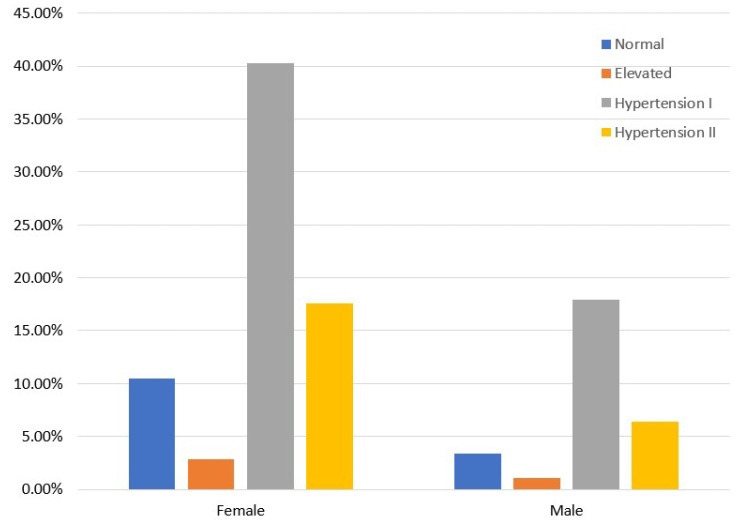
CVD participants’ distribution in terms of gender and blood pressure category in the balanced dataset.

**Figure 2:**
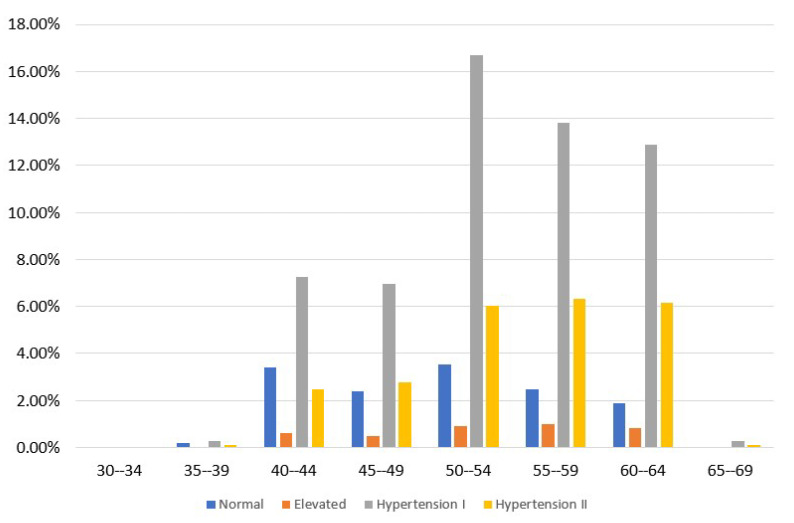
CVD participants’ distribution in terms of the age group and blood pressure category in the balanced dataset.

Moreover, Figure 1 isolates the CVD class and presents the distribution of the relevant instances concerning gender and blood pressure categories. Women with hypertension prevail against men, meaning that the former are more prone to occurring hypertension and CVD than the latter. Figure 2 demonstrates the distribution of participants per blood pressure category [45,46] and age group only for those who have been diagnosed with CVD. As we see, hypertension mainly concerns those older than 50 years, while a small portion of participants, 6 to 10%, occurs in the 40–44 and 45–49 age groups.

**Figure 3:**
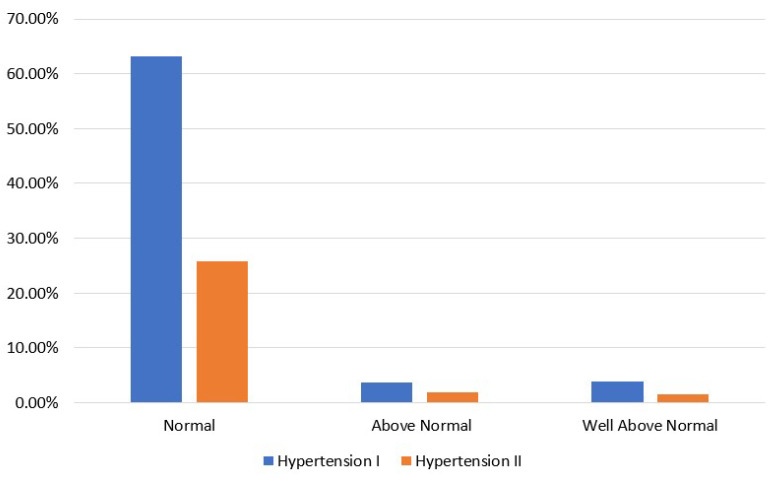
CVD participants’ distribution in terms of glucose level and hypertension classes in the balanced dataset.

Figure 3 shows the coexistence of hypertension and glucose levels in CVD participants. From relevant studies [59], it is known that hypertension is more frequent in CVD patients with diabetes in comparison with those who do not have diabetes. Moreover, CVD patients with hypertension are at greater risk of developing diabetes. However, in the current data, a small percentage of CVD patients has glucose levels well above normal and is hypertensive.

It should be noted that high blood pressure (BP), smoking habits, high abnormalities in glucose (which is associated with diabetes mellitus) and lipid levels (high cholesterol) are major risk factors for CVD, but they may be modified by proper interventions. Among these, high BP is the strongest causation and, in this study, it has a high prevalence of exposure in older than 50 years, and females [2].

### 2.3. Machine Learning Models for the CVD Risk Prediction

In this research article, we experimented with various ML models to uncover which one outperforms the others by evaluating their prediction performance. Specifically, we focused on Naive Bayes (NB) [60] and Logistic Regression (LR) [61], which are probabilistic classifiers. From Ensemble ML algorithms, Bagging [62], Rotation Forest (RotF) [63], AdaBoostM1 [64], Random Forest (RF) [65], Voting [66] and Stacking [67] were exploited. Moreover, a fully connected class of feedforward Artificial Neural Networks, i.e., Multilayer Perceptron (MLP) [68] and k-Nearest Neighbors (kNN) [69], a distance-based classifier, were evaluated. Finally, in Table 9, we illustrate the optimal parameters’ settings of the ML models that we experimented with.

### 2.4. Evaluation Metrics

In the context of ML models’ evaluation, we will consider Accuracy, Precision, Recall and Area Under Curve (AUC), which are the most frequently used in the relevant literature [70].

Accuracy indicates the overall classification performance by measuring the number of correctly predicted instances (CVD and non-CVD) in the whole data. Precision (or Positive Predicted Value) shows the ratio of positive subjects in relation to true and false positive subjects. In addition, Recall or sensitivity captures the ratio of subjects who had a CVD and are correctly classified (predicted) as positive, with respect to all positive subjects. The aforementioned metrics are defined as follows

(1) $$\begin{array}{c} \mathrm{Precision}=\frac{\mathrm{TP}}{\mathrm{TP}+\mathrm{FP}},\mathrm{Recall}=\frac{\mathrm{TP}}{\mathrm{TP}+\mathrm{FN}},\mathrm{Accuracy}=\frac{\mathrm{TN}+\mathrm{TP}}{\mathrm{TN}+\mathrm{TP}+\mathrm{FN}+\mathrm{FP}} \end{array}$$

where TP, TN, FP and FN stand for the True Positive, True Negative, False Positive and False Negative, respectively. Finally, AUC values will be recorded in order to evaluate the models’ efficiency. It varies between zero and one and is leveraged to identify the ML model with the highest probability of distinguishing CVD from non-CVD instances. When AUC attains one, this entails perfect discrimination among the instances of two classes. On the other hand, if all CVD instances are classified as non-CVD and vice versa the AUC equals 0.

## 3. Results

For the evaluation of our ML models, we relied on the Waikato Environment for Knowledge Analysis (Weka) [71]. In addition, the experiments were performed on a computer system with the following specifications: 11th generation Intel(R) Core(TM) i7-1165G7 @ 2.80GHz, RAM 16GB, Windows 11 Home, 64-bit OS and x64 processor. We applied 10-fold cross-validation to measure the models’ efficiency in the balanced dataset of 8734 instances after SMOTE.

In this research work, various ML models, such as NB, LR, MLP, kNN, RF, RotF, AdaBoostM1, Stacking, Voting and Bagging were evaluated by exploiting all of the above metrics. In Table 10, we illustrate the performance of the models under consideration before and after SMOTE with 10-fold cross-validation. As the results witness, class balancing (using SMOTE) favoured the models’ Precision and Recall performance metrics by at least 10% and a maximum of 36% depending on the classifier. From the results of our experiments, it is shown that the Stacking model, which has as base classifiers the Random Forest and Naive Bayes, and as a meta-classifier, the Logistic Regression indicated the best performance in both cases (No SMOTE and SMOTE) in comparison to the other models. In the SMOTE case, the Accuracy, Precision and Recall of Stacking were 87.8%, 88.3%, 88% and an AUC of 92.8%. In addition, the Rotation Forest, Bagging and Stacking models noted very proximal Accuracy of 87.2%, 87.6% and 87.8%, correspondingly. From the rest models, RF, Voting and AdaBoostM1 behaved similarly with an Accuracy equal to 86.6%, 86.7% and 86.8%. Moreover, neighbouring values in terms of Accuracy presented NB with 3NN and LR with MLP.

**Figure 4:**
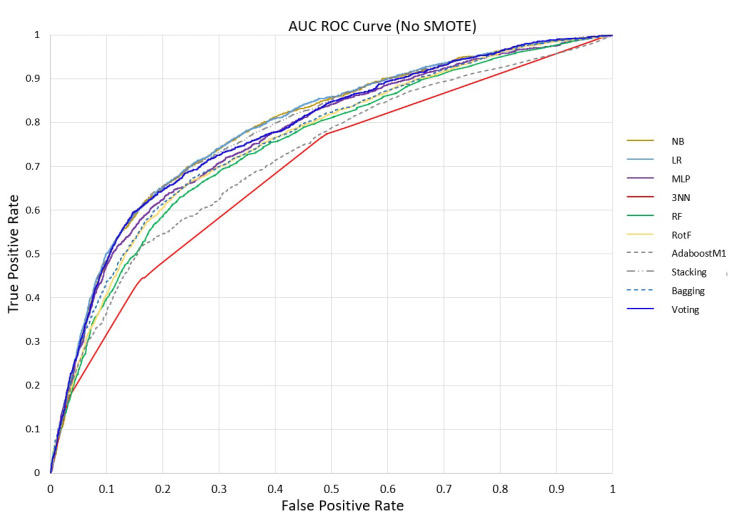
ML models AUC ROC Curve without SMOTE.

**Figure 5:**
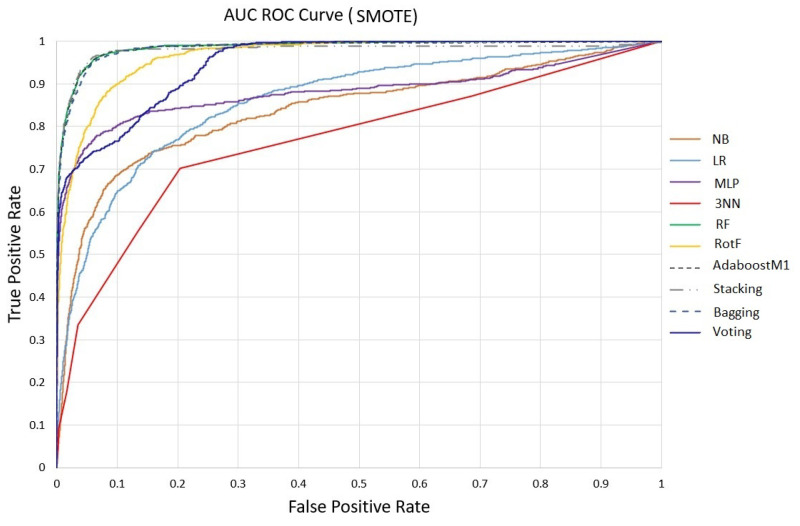
ML models AUC ROC Curve after SMOTE.

Regarding the AUC, percentages equal to or greater than 94% were achieved by RotF with 91.9%, Stacking with 92.1%, Voting with 91.7% and Bagging with 90.9%. Finally, for further validation of the models’ evaluation, in Figure 4 and Figure 5 we plot the AUC ROC curve of the proposed machine learning models without and with SMOTE, where the Stacking technique’s superiority is confirmed.

In concluding the evaluation of the proposed models, we have to point out some limitations concerning the dataset we relied on. First, it has not been acquired from a hospital, which could give us a more detailed profile of the subjects under consideration. In addition, in the current dataset, the amount of salt, alcohol and cigarette consumption is not available A diet with salt usage higher than the healthy limits is an important risk factor for hypertension development and, in turn, can lead to CVD [72]. Finally, as we have mentioned above, waist circumference is another feature lacking from the dataset under consideration. Let us recall that it is a strong indicator of abdominal obesity and may cause CVD.

## 4. Discussion

This section aims to provide a brief discussion of the most recent works on the prediction of CVDs using ML techniques and a variety of models.

The research community has applied various classifiers for CVDs prediction such as Logistic Regression [40,73,74], Support Vector Machine [40,73,74,75,76,77], Naive Bayes [40,73,75,76], Neural Networks (NN) [75], k-Nearest Neighbours [75,76], Decision trees (DT) [75], AdaBoost [77], Random Forests [40,73,74,75], Language Model (LM) [75] and Gradient Boosting (GB) [73,77,78]. In Table 11, we demonstrate a brief description of recent studies. The approaches listed in this table are compared, focusing on some key traits, i.e., the dataset, proposed model and performance metrics.

A CVD risk prediction model was developed in [73] using several classification methods such as SVM, Gradient Boosting, RF, NB and LR. The proposed approach aimed to provide awareness or diagnosis of CVD. The LR model achieved an accuracy of 86.5%. Moreover, a prospective study of 423,604 UK Biobank participants without CVD was made in [77]. The authors suggested an ML-based tool, called AutoPrognosis, for predicting CVD risk by experimenting with all or subsets of variables (from 473 available) to identify those that improve the accuracy of CVD risk prediction in the UK Biobank population. In addition, they validated their model by comparing its performance with the Framingham Risk Score, Cox proportional hazards model, and some standard ML models (linear SVM, AdaBoost, and Gradient Boosting Machines).

Another interesting and recent approach is presented in [79]. More specifically, a neuro-fuzzy (NF) decision support system (namely, a combination of neural networks with fuzzy logic [80]) is proposed for learning predictive models in form of fuzzy rules, to assess cardiovascular risk. The level of cardiovascular risk is identified by selecting important signs for CVD patients, such as the heart rate (HR), breathing rate (BR), blood oxygen saturation (SpO2) and lips colour. Among the investigated fuzzy models, the NF after data oversampling achieved the maximum accuracy of 91%. The NF system has also prevailed in comparison with the standard models (RF, MLP, DT, XGBoost).

From now on, we will focus on studies that exploited the same dataset [81] as the current study. More specifically, in [74], the authors used the correlation coefficient for feature extraction before applying ML in order to develop prediction models for CVD. The results showed that the SVM achieved an AUC of 78.84% under 5-fold cross-validation, which indicated better performance in comparison to the other models (namely, LR and RF).

Furthermore, on [75], the authors proposed a new method that aimed to find significant features by applying ML techniques resulting in improvement of the CVD predictive accuracy. The prediction model was developed assuming different combinations of features and several known classification models (SVM, RF, NB, ANN, kNN, LM). The experiment was repeated with the aforementioned ML techniques using 13 attributes. They produced an enhanced performance accuracy level of 88.7% through the hybrid random forest using a linear model (HRFLM) for CVDs prediction.

In [76], the authors’ purpose was to enhance the performance of ML models by applying data preprocessing through normalization that fills up missing data with the mean value of each feature instead of ignoring them. For the evaluation, kNN, NB and SVM models were compared. The linear kernel SVM prevailed with an accuracy of 86.8% assuming an 80:20 training and testing ratio, respectively. Moreover, the article [78], proposed a recursive feature elimination-based gradient boosting algorithm to achieve an accurate heart disease prediction. The proposed model achieved an accuracy of 89.7% and an AUC equal to 84%.

In addition, in the recently published research work [40] that exploited the same dataset as the four previous studies, the selected ML models were trained and tested without data preprocessing to tackle empty and invalid data. Comparing the current outcomes with the respective ones in [40], we observed that NB noted an essential increase concerning all performance metrics, remaining the model with the highest performance improvement. More specifically, NB Accuracy and Recall boosted from 59.6% to 72.2% while AUC noted an increase from 69.4% to 78.1%. Moreover, the LR model performed better, but the increase was small: (i) Accuracy and Recall raised from 72.1% to 72.7% and (ii) AUC increased from 78.4% to 79.1%. As for the RF model, we see that the Accuracy, Recall (from 70.9% to 86.2% ) and AUC (from 76.6% to 91.9%) were enhanced by 15.3%. Isolating AUC has revealed the high inherent ability of the tree model to discriminate between the diseased and healthy participants.

Here, we applied 10-fold cross-validation to the dataset that resulted after cleaning and then class balancing (using SMOTE) to tackle the non-uniform distribution of the subjects in the two states (CVD, non-CVD). Note that in the current study, we resorted to more efficient schemes to design the desired classification models, with an emphasis on ensemble techniques. In addition, we further validated the expected performance of ensemble models with a graphical illustration of the AUC ROC curves. To sum up, comparing the performance of the suggested models in previous works with the ones applied here, the newly trained and tested stacking ensemble classifier prevailed, achieving the highest AUC of 98.2%, although the Accuracy was 87.8%.

## 5. Conclusions

Recent developments in artificial intelligence (AI) and ML have facilitated the identification of individuals at high risk for a disease manifestation, and they are exploited in the present study to design a methodology for the risk prediction of cardiovascular disease occurrence based on several risk factors. The suggested approach could support healthcare professionals and clinical experts in their efforts to prevent the severe compilations of CVD in both individual patients and at a population level. Through data analysis, we tried to understand and explore the association of features with CVD and discover hidden patterns concerning the signs related to CVD.

Moreover, through risk factors monitoring and analysis, personalized guidelines and interventions can be suggested to prevent CVD occurrence. Medical experts can benefit from ML models to regularly reassess the underlying risk. In addition, even if CVD occurs, they can provide patients with novel guidelines and treatments based on individual patient characteristics that may enhance their daily life, increase life expectancy and restrict mortality.

In this research work, plenty of ML models were evaluated considering a mixture of anthropometric and biochemical data acquired by a non-invasive process. Actually, it is a dataset that captures the most relevant factors (systolic and diastolic blood pressure) and human habits that feed the occurrence of CVD. From the analysis, we demonstrated that the application of data preprocessing through cleaning and class balancing is an essential step for the design of efficient and accurate models to quantify the CVD risk and, thus, achieve a performance boost. Moreover, the experiments’ results showed that the Stacking model outperformed the other models, reaching an Accuracy of 87.8%, Precision of 88.3%, Recall of 88% and AUC of 98.2% after SMOTE with 10-fold cross-validation.

In future work, we aim to experiment with data that, except for the current features, also assume waist circumference, amount of salt, alcohol and tobacco and reevaluate the predictive ability of the current models. A challenging extension is the adoption of the Shapley Additive exPlanations (SHAP) [82] method for measuring feature importance and interpreting the machine learning models’ performance. SHAP is a game-theoretic technique for explaining the output of any machine-learning model [83]. Finally, deep learning models will be investigated and tested in CVD occurrence. 

## Figures and Tables

**Table 1:** Numerical and nominal features’ description in the unbalanced dataset.

Numerical Attribute	Description		
	**Min**	**Max**	**Mean ± StdDev**
**Age**	30	65	52.73 ± 6.86
**BMI**	15.36	58.59	27.12 ± 5.11
**Sys BP**	70	220	123.8 ± 15.3
**Dias BP**	40	150	80.25 ± 9.1
**Nominal Attribute**	**Description**		
**Gender**	Men 2184 (34.6%) Women 4127 (65.4%)		
**Glucose**	(85.6%) normal (7.4%) above normal (7%) well above normal		
**Smoke**	Yes (9.2%)		
**Alcohol Intake**	Yes (5.4%)		
**Physical Activity**	Yes (80.1%)		
**Total Cholesterol**	(78.1%) normal (12.7%) above normal (9.2%) well above normal		

**Table 2:** Features ranking before and after class balancing.

Random Forest				Gain Ratio				Information Gain			
SMOTE		No SMOTE		SMOTE		No SMOTE		SMOTE		No SMOTE	
Attribute	Rank	Attribute	Rank	Attribute	Rank	Attribute	Rank	Attribute	Rank	Attribute	Rank
Age	0.253	SysBP	0.23907	SysBP	0.07589	SysBP	0.07243	SysBP	0.16307	SysBP	0.14602
SysBP	0.2426	DiasBP	0.17963	DiasBP	0.06083	DiasBP	0.05732	DiasBP	0.10531	DiasBP	0.09216
BMI	0.1897	Age	0.12714	Age	0.03809	Cholesterol	0.03947	Age	0.08847	Cholesterol	0.03837
DiasBP	0.1893	Cholesterol	0.09492	Cholesterol	0.02763	Age	0.01894	BMI	0.03646	Age	0.03511
Cholesterol	0.0574	BMI	0.04311	Smoke	0.02654	BMI	0.01489	Cholesterol	0.02615	BMI	0.02709
Gender	0.0519	Glucose	0.02689	BMI	0.0177	Glucose	0.00806	Gender	0.01067	Glucose	0.00594
Physical activity	0.03550	Physical activity	0.00931	Alcohol intake	0.01749	Physical activity	0.00157	Smoke	0.00935	Physical activity	0.00113
Smoke	0.0263	Smoke	0.00135	Physical activity	0.0146	Smoke	0.00026	Physical activity	0.00887	Smoke	0.00012
Alcohol intake	0.0144	Alcohol intake	−0.00339	Gender	0.01231	Gender	0.00006	Alcohol intake	0.00416	Gender	0.00006
Glucose	0.012	Gender	−0.00467	Glucose	0.00293	Alcohol intake	0.00002	Glucose	0.00178	Alcohol intake	0.000006

**Table 3:** Statistical characteristics in the balanced data.

Features	Min	Max	Mean ± Std
**Age**	30	65	53.36 ± 6.76
**BMI**	15.36	58.59	27.48 ± 5.17
**Sys BP**	70	220	126.46 ± 16.33
**Dias BP**	40	150	81.44 ± 9.39

**Table 4:** Participants’ distribution per age group and gender type in the balanced dataset.

Age Groups	Non-CVD	CVD
30–34	0.01%	0.00%
35–39	0.53%	0.10%
40–44	9.53%	4.24%
45–49	7.05%	5.60%
50–54	15.14%	12.06%
55–59	10.03%	13.57%
60–64	7.51%	14.24%
65–69	0.21%	0.19%
**Gender**	**Non-CVD**	**CVD**
Female	32.84%	38.33%
Male	17.16%	11.67%

**Table 5:** Participants’ distribution per BMI class and physical activity in the balanced dataset.

BMI Classes	Non-CVD	CVD
Underweight BMI <18.5	0.70%	0.14%
Healthy 18.5≤ BMI < 25	21.71%	14.79%
Overweight 25≤ BMI < 30	17.99%	19.45%
Obese I 30≤ BMI <35	6.90%	9.67%
Obese II 35≤ BMI <40	2.06%	4.21%
Obese III BMI ≥40	0.64%	1.73%
**Physical Activity**	**Non-CVD**	**CVD**
No	9.41%	5.48%
Yes	40.59%	44.52%

**Table 6:** Participants’ distribution per cholesterol and glucose level in the balanced dataset.

Cholesterol	Non-CVD	CVD
Normal	41.88%	37.29%
Above Normal	5.69%	4.59%
Well Above Normal	2.43%	8.12%
**Glucose**	**Non-CVD**	**CVD**
Normal	43.85%	45.20%
Above Normal	3.33%	2.24%
Well Above Normal	2.82%	2.55%

**Table 7:** Participants’ distribution per smoking and alcohol status in the balanced dataset.

Smoke	Non-CVD	CVD
No	45.53%	45.65%
Yes	4.48%	4.36%
**Alcohol**	**Non-CVD**	**CVD**
No	45.28%	48.08%
Yes	4.72%	1.92%

**Table 8:** Participants’ distribution in terms of blood pressure category in the balanced dataset.

Sys/Dias Blood Pressure Categories [58]	Non-CVD	CVD
**Normal** Sys BP <120 and Dias BP <80	10.37%	3.53%
**Elevated** 120< Sys BP <129 and Dias BP <80	2.61%	1.32%
**Hypertension I** 130< Sys BP <139 or 80< Dias BP <89	32.24%	25.96%
**Hypertension II** Sys BP ≥139 or Dias BP ≥90	4.77%	19.20%

**Table 9:** Machine Learning Models’ Settings.

Models	Parameters
**NB**	useKernelEstimator: False useSupervisedDiscretization: True
**LR**	ridge = $10^{-8}$ useConjugateGradientDescent: True
**MLP**	learning rate = 0.1 momentum = 0.2 training time = 200
**kNN**	k = 3 Search Algorithm: LinearNNSearch with Euclidean cross-validate = True
**RF**	breakTiesRadomly: True numIterations = 500 storeOutOfBagPredictions: True
**RotF**	classifier: Random Forest numberOfGroups: True projectionFilter: PrincipalComponents
**AdaBoostM1**	classifier: Random Forest resume: True useResampling: True
**Stacking**	classifiers: Random Forest and Naive Bayes metaClassifier: Logistic Regression
**Voting**	classifiers: Random Forest and Naive Bayes combinationRule: average of probabilities
**Bagging**	classifiers: Random Forest printClassifiers: True storeOutOfBagPredictions: True

**Table 10:** Performance Evaluation of ML models before and after SMOTE.

	Accuracy		Precision		Recall		AUC	
	No SMOTE	SMOTE	No SMOTE	SMOTE	No SMOTE	SMOTE	No SMOTE	SMOTE
**NB**	0.771	0.836	0.648	0.849	0.560	0.791	0.787	0.866
**LR**	0.772	0.846	0.706	0.855	0.444	0.799	0.789	0.880
**MLP**	0.768	0.840	0.656	0.858	0.519	0.806	0.771	0.894
**3NN**	0.714	0.833	0.544	0.801	0.446	0.807	0.695	0.811
**RF**	0.740	0.866	0.588	0.877	0.522	0.874	0.749	0.977
**RotF**	0.752	0.872	0.614	0.875	0.527	0.860	0.759	0.940
**AdaBoostM1**	0.738	0.868	0.584	0.876	0.521	0.871	0.724	0.976
**Stacking**	0.776	0.878	0.676	0.883	0.560	0.880	0.786	0.982
**Bagging**	0.753	0.876	0.619	0.878	0.520	0.876	0.763	0.975
**Voting**	0.775	0.867	0.660	0.834	0.557	0.838	0.781	0.946

**Table 11:** An overview of recent studies for CVD risk prediction.

Reference	Dataset	Proposed Model	Performance
[40]	[81]	Logistic Regression	AUC 78.4% Accuracy 72.1%
[73]	Long Beach VA heart disease database	Logistic Regression	Accuracy 86.5%
[74]	[81]	SVM	AUC 78.84%
[75]	[81]	Hybrid Random Forest with a linear model (HRFLM)	Accuracy 88.7%
[76]	[81]	SVM (linear kernel)	Accuracy 86.8%
[77]	UK Biobank	AutoPrognosis model	AUC 77.4%
[78]	[81]	Gradient Boosting algorithm	AUC 84% Accuracy 89.7%
[79]	Not Publicy Available	Neuro-Fuzzy model	Accuracy 91%

## Data Availability

Not applicable.
